# Mental health and help-seeking behavior among at-risk university students in Germany: A descriptive analysis

**DOI:** 10.1371/journal.pmen.0000576

**Published:** 2026-03-20

**Authors:** Sarah-Lena Klemm, Stefanie Dreiack, Aneliana da Silva Prado, Konrad Endres, Elisabeth Kohls, Christine Rummel-Kluge

**Affiliations:** 1 Department of Psychiatry and Psychotherapy, Medical Faculty, Leipzig University, Leipzig, Germany; 2 Office for Equal Opportunities Saxony, Leipzig, Germany; 3 Department of Psychiatry and Psychotherapy, University Leipzig Medical Center, Leipzig University, Leipzig, Germany; Latrobe Regional Hospital, AUSTRALIA

## Abstract

University students mental health has become a growing concern worldwide with significant disparities among at-risk groups such as students with chronic illnesses, mental health diagnoses, migration histories, diverse gender identities, and parental responsibilities. These at-risk groups face unique challenges and increased psychological distress, highlighting the need for further research to better understand their specific needs and to shed a light on broader societal issues such as discrimination and stigmatization, thereby informing the development of tailored support measures in higher education settings. These disparities can also be interpreted through Andersen’s Behavioral Model, which posits that help-seeking behavior is shaped by predisposing, enabling, and need-related factors. The analyses includes data that were collected between April and May 2022 from *N* = 5474 students from Saxony, Germany. Via an online survey, variables were assessed using standardized questionnaires on depressive symptoms, anxiety, stress and perceived social support. Further, students were asked on their professional and university help seeking-behavior. Across all five pre-defined at-risk groups of university students, individuals belonging to one of the at-risk groups showed worse mental health outcomes than their respective comparison group. Only for parental students, a different pattern was found, with lower psychopathology and higher perceived social support than non- parents. Regarding the use and knowledge of professional and university help offers, the respective subgroups showed different preferences and knowledge. Viewed through Andersen’s Behavioral Model, these findings suggest that elevated psychological distress reflects higher need factors, whereas differences in perceived social support represent variations in enabling factors that may shape help-seeking patterns and service utilization. These findings highlight the urgent need for flexible, low-threshold support options such as online interventions, which can effectively complement traditional counseling services in universities.

Study registration

DRKS00030697.

## Introduction

There is growing evidence indicating the pressing need to address university students mental health worldwide, especially since the COVID-19 outbreak [1–11]. Although students faced similar outcomes of the COVID-19 pandemic - such as social distancing, universities closure, online teaching – to name a few, they were affected differently, as contextual circumstances and (inter)personal factors varied along the availability of support.

While an overall trend of increased stress, anxiety, and depression was observed among students during the pandemic [10], some groups reported a greater impact on their mental health and well-being. Several groups of university students have been identified as at-risk populations in higher education settings, including those with special educational needs and disabilities [12,13], students with a chronic somatic disorder [14], with lower socioeconomic status [15–18], and students with a mental disorder diagnosis or who reported mental health issues [19], for instance.

To gain better understanding why these at-risk groups may not only differ in their mental health status but also in their help-seeking behavior, it is useful to consider theoretical models of health service use. A widely applied framework is the [20], which proposes that service utilization is shaped by predisposing factors (e.g., gender, migration background), enabling factors (e.g., financial resources) and need factors (e.g., symptom severity). More recent adaptions for marginalized young adults [21] emphezise additional factors such as minority stress, stigma and discrimination. Applying the Andersen Behavioral Model helps to explain why different at-risk groups may vary in their intention and likelihood to use professional or university help-services. In help-seeking research, “intention” refers to a student’s planned willingness to seek support, whereas “likelihood” reflects their perceived probability of actual doing so. Clarifying this conceptual distinction is crucial, as both constructs relate differently to help-seeking among at-risk student groups.

Gender is one of the most discussed predisposing factors, while gender disparity among university students has been also reported [15,16,19]. While female students often report greater levels of stress than to their male counterparts [22], sexual and gender minority (SGM) individuals face chronic stressors related to their marginalized identities- including stigma, prejudice and discrimination- which can lead to psychological distress and poorer mental health outcomes [15,23]. For example, in a sample of over 480.000 U.S. college and university students, SGM students were more than three times more likely to report depression than non-SGM students [24]. In addition, there is still limited research on the specific needs of SMG students in terms of their identity, particularly concerning mental health in university settings.

Although some studies found no significant difference in overall mental health between students who are parents and those who are not [13,25], student parenthood is often associated with financial challenges and substantial additional responsibilities [26]. Single-parenting students, in particular, experience a higher prevalence of mental health problems and report twice as many suicide attempts than other college students [27].

At-risk groups such as international students and students with a migration background have also been associated with increased psychological distress, and reduced well-being compared to their domestic counterparts [28,29].

These aforementioned groups have been consistently associated with a poorer mental health outcomes, increased psychological distress, and higher rates of depression and anxiety during the pandemic [3,13,15,19], highlighting the importance of identifying and addressing the inequities that place them at risk [30]. Given these significant disparities and unique challenges, it is essential to assess the mental health status of these students to develop targeted support services and promote their well-being in academic settings, while also addressing the health inequities they face. This raises questions of whether future support for well-being should target these students populations specifically [31,32], making it essential to accurately describe and understand the mental health status of these groups in higher education settings.

In help-seeking research, “intention” refers to a student’s planned willingness to seek support, whereas “likelihood” reflects their perceived probability of actual doing so. Clarifying this conceptual distinction is crucial, as both constructs relate differently to help-seeking among at-risk student groups.

Due to the pressing need to develop and improve prevention and promotion strategies, as well as support measures more tailored to these populations [8], this study aimed to investigate the mental health and help-seeking behavior among at-risk university students (pre-defined by literature, see methods section) and determine the kind of counseling and support they would need.

## Materials and methods

### Ethics statement

The ethics committee of Leipzig University granted approval for this study on November 11^th^, 2021 (file reference: 509/21-ek). All participants provided online informed consent before participating via an opt-in function.

### Participants and procedure

Data for this exploratory cross-sectional study were collected April 11^th^ to May 27^th^, 2022 among students enrolled at six universities in the Saxony, Germany, Recruitment was carried out via the universities central communication channels (e.g., mailing lists, social media). All enrolled students were eligible to participate regardless of study program, degree level, or nationality. Participation was anonymous, voluntary and uncompensated. The survey was available in German and English to reduce language barriers for international students. A total of N = 5.474 students provided complete data and were included in the analyses. Cases with incomplete data or key mental health variables were excluded from the analyses. Given the large sample size, the number of excluded cases was minimal and unlikely to bias the results. The ethics committee of Leipzig University granted approval for this study on November 11^th^, 2021 (file reference: 509/21-ek).

### Measures

#### Quantitative analyses.

**Demographic information:** The following demographic information was assessed: age, gender (female, male, diverse), parental status (being a parent), and migration history (self, parents or no migration history). Additionally, participants were asked about having a chronic somatic disorder (“Do you suffer from a (chronic) physical disease?”) and whether they had been diagnosed with a mental disorder in the past (“Have you been diagnosed with mental illness/disorder in the past?”) with” answer options of “yes” or “no.

**Mental health measures:** The Patient-Health-Questionnaire-9 (PHQ-9) was used to assess depressive symptoms [33]. The questionnaire consists of nine items, each rated on a 4-point Likert scale ranging from 0 = “not at all” to 3 = “nearly every day.” A sum score is computed, with higher scores indicating more severe depressive symptoms.

The Generalized Anxiety Disorder scale (GAD-7) [34] was used to assess symptoms of generalized anxiety disorder. The questionnaire consists of seven items with a 4-point rating scale ranging from 0 = “not at all” to 4 = “nearly every day,” assessing symptoms experienced over the past two weeks. A sum score is computed, with higher scores indicating greater symptom severity. Cases of likely generalized anxiety disorder are identified when the sum score exceeds 10 ([34];[35]).

**Help-seeking behavior, and use of mental health services:** To access students’ help-seeking intentions, awareness of mental health support services, and actual use of professional and university offers, four different questionnaires were used. In addition, students were asked about their user intention of hypothetical offers and their perceptions of an optimal online help-seeking program for mental health problems. The questionnaires were used as follows:

**Questionnaire for intention to use professional help-services:** In this questionnaire, students were asked what kind of professional service they would use if they experienced mental health problems. The questionnaire included 7 items: psychiatrist, psychotherapist, psychosocial counseling, alternative/non-medical practitioner for psychotherapy, inpatient treatment in a psychiatric/somatic clinic, online help services, and self-help books/guides. For five of these items, participants indicated the likelihood of using the service on a scale ranging from 0 = “very unlikely” and 3 = “very likely.” Additionally, participants could also specify a service and its likelihood of use in a free text field. If participants indicated at least one of the services, it was interpreted as having the intention to use a professional help-service in case of experiencing mental health problems. Furthermore, answer options were binary coded, where a score of 1 or higher on the scale was considered to reflect the participants’ likelihood of using the respective service.

**Question for actual use of professional help-services:** In this questionnaire, participants were asked which mental health services they had already used in the past including psychiatrist, psychologist, psychosocial counseling, inpatient stay in a psychiatric/psychosomatic clinic, and online help services. The response options were “I have used this service before” and “I have used this service last year.” If neither option was selected, the answer was considered as no past use of professional help. Responses indicating service use either before or within the last year were combined and interpreted as actual use of professional help-services.

**Questionnaire on awareness and use of universities’ support services:** In these questionnaires, participants were asked about university support services and which ones they had already used. The following items were listed as response options: central student advisory service, psychological counseling, counselling service of students with children, workshops on coping with stress, exam anxiety or mindfulness, and orientation days. For each item, students were asked whether they were aware of the service, with three response options: “yes”, “no,” and “I don't know”. A response of “Yes” was interpreted as awareness of the service, while “no” and “I don’t know” were interpreted as unawareness. Following this, students were asked whether they had used the service, with three response options: “once”, “multiple times” or “never.”;

**Potential use of other universities’ support services:** To explore student’s acceptance and potential use of support services not previously mentioned, participants were asked whether they would use any of the following options: face-to-face individual counseling, face-to-face group counseling, telephone counselling, chat counselling, e-mail-counseling, written counseling (brochures, literature), information via homepages, and forums/blogs.

**Students’ perceptions of an optimal online program for receiving support in mental health issues:** To gain a better understanding of student’s perceptions, attitudes, and preferences regarding a a hypothetical online program for mental health support, participants were asked to indicate, using a “yes” or “no” answer options which seven given aspects they considered relevant: professional expertise, anonymity, short notice contact option, reference person, good user experience, free of charge, and no data exchange between health insurance and program.

**Socioemotional outcomes:** The Perceived Stress Scale (PSS-4) was used to asses participants’ perceived stress [36]. The questionnaire consists of four items rated on a 5-point Likert scale ranging from 0 = “never,” to 4 = “very often.” A total sum score is computed, with higher scores indicating higher perceived stress.

The ENRICHD Social Support Inventory (ESSI) was used to assess perceived social support [37,38]. The instrument consists of five items rated on a 5-point Likert scale ranging from 1 = “none of the time” to 5 = “all of the time.” A total sum score is computed, with higher scores indicating greater perceived social support.

**Application of the Andersen model:** In accordance with Andersen`s Model of Health Service Use, study variables were conceptually assigned to predisposing, enabling, and need-related factors. Predisposing factors included sociodemographic characteristics such as age, gender identity, parental status, migration history, and international student status. Enabling factors included social support (ESSI) as well as awareness of and actual use of university-based mental health help offers. Need factors were operationalized through indicators of psychological distress, including depressive symptoms (PHQ-9), anxiety symptoms (GAD-7), perceived stress (PSS-4) as well as self-reported diagnoses of mental health and chronic somatic disorders. The Andersen model was used to interpret subgroup differences in mental health outcomes and help-seeking intentions and behaviors.

**Pre-defined at-risk subgroups of university students:** In line with the aim of this study, participants were assigned to five pre-defined subgroups based on prior literature (see introduction) and developed in scientific collaboration with the “Office for Equal Opportunities Saxony”, which promotes equality among diverse student groups. This classification was further supported by extended literature research on at-risk university student populations. The at-risk subgroups for this analysis were as follows: parental students, students with chronic diseases, students diagnosed with a mental disorder, students with a migration history (i.e., whose parents were migrants) or international students, and gender subgroups (male, female, diverse).

### Statistical analysis

Statistical analyses were performed using IBM SPSS Statistics version 29.0. A two-tailed α = 0.05 significance level was used for statistical testing. Descriptive statistics for demographic information (age, gender, being a parent, chronic somatic illnesses, mental disorders) and mental health outcomes (depressive symptoms, PHQ-9; anxiety, GAD-7), socioemotional outcomes (perceived social support, ESSI; perceived stress, PSS-4) were computed separately for each subgroup.

Differences between subgroups in categorical variables- including demographic characteristics, help-seeking behavior, use of mental health services, intention to use and actual use of professional help services, awareness and use of universities’ support services, and students’ perception of an optimal online program for mental health support- were analyzed using χ^2^-tests. To estimate effect sizes, the φ-coefficient was used. When the contingency table exceeded a 2 × 2 format, Cramér’s V (φc) was applied. Effect sizes were interpreted as follows: φ or φc = 0.10 as small, φ or φc = 0.30 as medium, and φ or φc = 0.50 as large [39].

Subgroup differences in continuous dependent variables (age, perceived stress, perceived social support, depressive symptoms, and anxiety) were analyzed using a MANOVA. The effect size was interpreted as small at η² = 0.001, medium at η² = 0.06, and large at η² = 0.14 [39]. Bonferroni correction was applied to adjust for multiple testing.

#### Qualitative analyses.

**To complement the quantitative data analyses, the study included a** free text item asking participants: “What kind of service would you use if you had mental health problems?” The aim of the qualitative analyses was to explore students’ preferred source of support and to identify themes that may not be fully captured by predefined response options.

Qualitative data analysis was performed by using MAXQDA qualitative software (version 2024.0.0). The qualitative analysis followed Mayring’s summarizing content analysis method [40]. All responses were read repeatedly by the first author to gain an overall understanding of the material.

A coding dictionary was developed to categorize recurring responses from the open-text fields. The resulting codes reflected the various approaches to mental health help-seeking described by participants and were refined in several iterative rounds. To ensure that all responses were considered, the coding categories were designed to be as few as possible, but as many as necessary. Based on the final coding manual, all statements were coded by one author. To ensure validity, inter-rater reliability was assessed. A random subset comprising 25% of the dataset was double-coded by a second, unbiased researcher. Discrepancies were discussed and resolved through consensus. As the qualitative component served an explorative purpose and aimed to provide a descriptive overview of student’s perspectives, no formal Cohen’s kappa value is reported, After coding, frequencies of all final coding categories were descriptively analyzed.

## Results

### Participants

Of the *N* = 5,474 participants in the survey, *n =* 316 (5.7%) indicated they had least one child and therefor belonged to subgroup parental students. Additionally, *n* = 933 participants reported having a chronic somatic illness, while *n* = 1,239 indicated they had received a diagnosis of a mental disorder. Regarding migration background, n = 4,774 participants reported no migration background, whereas n = 393 reported a migration background (i.e., either they or their parents had a migration background). Furthermore, n = 307 were international students (i.e., participants who indicated a migration background but whose parents did not). Finally, the gender subgroup analysis showed that n = 3,775 participants identified as female, n = 1,599 as male, and n = 100 as gender diverse.

### Quantitative analyses

#### Group 1 at-risk university students: Parental students.

To begin with, there was a comparison between parental students and their non-parental peers to examine whether parenthood is associated with different mental health outcomes and patterns of service use. There were *n =* 316 (5.7%) students who indicated that they had at least one child in the study. Most parental students identified as female (*n =* 233, 73.7%), while *n =* 77 (24.4%) identified as male, and 1.9% (n = 6) identified their gender as diverse. The age of parental student’s ranged from 19 to 56 years, with a mean age of M = 34 (SD = 7.56). In comparison, non-parental students (*n =* 5,158, 94.3%) had an average mean age of M = 23.7 (.SD = 3.73). Moreover, *n =* 57 (18.0%) of parental students indicated they had a chronical disease, while over a quarter, *n =* 85 (26.9%) indicated they had been previously diagnosed with a mental health disorder. Parental students showed lower levels of stress (PSS4), depression (PHQ-9), and anxiety (GAD7) compared to non-parental students (see Table 1).

**Table 1:** Group differences between parental students and non-parental students in regards of awareness and actual use of (mental health and university) support services (N = 5,474).

Variable, *n* (%)	Parental students (*n* = 316)	Non-parental students (*n* = 5,158)	Test	*p*	Effect size
Psychopathology					
Depressive Symptoms (PHQ-9)	7.21 (5.15)^b^	8.41 (5.53)^a^	*F*(1, 5474) = 14.25	**<.001**	η² = .00
Gender, *n* (%)			χ^2^(2) = 3.81	.149	ϕ_c_ = .03
Female	233 (73.7)	3,542 (68.7)			
Male	77 (22.4)	1,522 (29.5)			
Diverse	6 (1.9)	94 (1.8)			
Age, *M* (*SD*)	34.16 (7.56)	23.7 (3.73)	F(1, 5474) = 15.565	**<.001**	ϕ_c_ = .003
Past usage of student advisory service, *n* (%)	99 (31.3)	896 (17.4)	χ^2^(1) = 39.0	**<.001**	ϕ = -.08
Past usage, counseling service for parental students, *n (%)*	94 (29.7)	21 (0.4)	χ2(1) = 1246.2	**<.001**	ϕ = -.47
Potential use of, forums/ blogs, *n (%)*	60 (19)	1,395 (27)	χ2(1) = 9.90	**.002**	ϕ = .04
Importance of good user experience in an online-support offer, *n (%)*	117 (37)	2,371 (46)	χ2(1) =.9.60	**.002**	ϕ = .04

*Notes.* PSS-4: Perceived Stress Scale; ENRICHD Social Support Inventory (ESSI); Depressive symptoms were measured with Patient-Health-Questionnaire-9 (PHQ-9) and Anxiety was measured with Generalized Anxiety Disorder scale (GAD-7).

There was no significant statistical differences in perceived social support between the groups. In regards of user intention of professional mental health support (psychiatrist, psychotherapist, psychosocial counseling, clinic, online groups), parental students did not differ from non-parental students. Parental students’ awareness of their universities counseling services and other supportive offers did not differ from non-parental students. Similar, the actual use of university support offers did only differ slightly between both groups: parental students used their universities student advisory service and the counseling service for parents significantly more often. There was no significant difference between parental students and non-parental students in all other offered university services (all *p* > .002, see Table 1).

When asked which university offers they would potentially use, parental-students did not differ from non-parental students, besides the fact that non-parental students would use forums or blogs significant more often for mental health support. When being asked for attitudes and inputs for an ideal online support program, non-parental students presented a higher value onto the program offering a good user experience in general (*p* = .002).

Finally, parental-students did not differ from their non-parental counterparts in regards their attitudes Table 1. Group differences between parental students and non-parental students in regards of awareness and actual use of (mental health and university) support services (N = 5,474).

Taken together, these results converge to suggest that although parental students report slightly lower levels of stress and depressive symptoms, their awareness and actual use of professional mental health and university support services are largely comparable to those of non-parental-students.

#### Group 2: Students diagnosed with a chronic somatic disorder.

Group 2 consisted of *n* = 933 (17.0%) students who indicated they had been previously diagnosed with a chronic somatic disorder. Most of these students identified themselves as female (*n* = 630; 67.5%), while *n* = 223 (23.9%) identified as male, and *n* = 30 (3.2%) as gender diverse. There was no statistical difference in age between students with a chronic somatic disorder students (*M* = 24.7%, *SD* = 5.63) and their counterparts (*M* = 23.5, *SD* = 4.95).

Among students with a chronic somatic disorder, n = 305 (32.7%) reported a previous diagnosis of a mental disorder. Additionally, n = 934 (20.6%) students without a chronic somatic disorder reported a diagnosis of a mental disorder. This difference between the groups was significant (p < .001).Students with a chronic somatic disorder reported higher levels of depression, anxiety, and stress, as well as lower perceived social support (all p < .001), with small effect sizes (see Table 2). Their intention to seek professional help did not differ significantly from students without a chronic somatic disorder (p = .586). However, students with a chronic somatic disorder had used professional mental health services significantly more often in the past (p < .001).Regarding awareness of university services, students with and without chronic somatic disorders did not differ significantly. Nevertheless, students with chronic somatic disorders used the student advisory services and psychosocial student services significantly more often than students without chronic somatic disorders (p < .001).For all other variables, no statistically significant differences were found (all p > .002 after Bonferroni correction).

**Table 2:** Group comparison between students with a chronic somatic disorder and students without a chronic somatic disorder in regards of user intention and actual usage of (mental health and university) support services.

Variable, *M* (SD)	Students diagnosed with a chronic somatic disorder (*n* = 933)	Students without a chronic somatic disorder (*n* = 4,541)	Test	*p*	Effect size
Psychopathology					
Stress (PSS4)	7.77 (3.15)	7.12 (3.15)	*F*(1,5474) = 32.64	**<.001**	η² = .00
Social Support (ESSI)	20.23 (4.07)	20.80 (4.07)	*F*(1,5474) = 14.92	**<.001**	η² = .00
Depressive Symptoms (PHQ-9)	9.69 (5.90)	8.07 (5.39)	*F*(1,5474) = 68.40	**<.001**	η² = .01
Anxiety (GAD7)	8.75 (5.06)	7.55 (4.70)	*F*(1,5474) = 49.29	**<.001**	η² = .00
Variable, *n* (%)					
Gender, *n* (%)			χ^2^(2) = 25.23	**<.001**	ϕ = .07
Female	680 (72.9)	3,095 (68.2)			
Male	223 (23.9)	1,376 (30.3)			
Diverse	30 (3.2)	70 (1.5)			
Age, *M* (*SD*)	24.7 (5.63)	23.5 (4.95)	F(1,5474) = 2228.132	**<.001**	ϕ = .289
Past use professional health care, *n* (%)	502 (53.8)	1,813 (39.9)	χ^2^(1) = 61.09	**<.001**	ϕ = -.11
Past usage, student advisory service, *n* (%)	216 (23.2)	779 (17.2)	χ^2^(1) = 18.71	**<.001**	ϕ = **-**.06
Past usage, psychosocial student counseling, *n (%)*	145 (15.5)	477 (10.5)	χ2(1) = 19.50	**<.001**	ϕ = -.06

Notes. PSS-4: Perceived Stress Scale; ENRICHD Social Support Inventory (ESSI); Depressive symptoms were measured with Patient-Health-Questionnaire-9 (PHQ-9) and Anxiety was measured with Generalized Anxiety Disorder scale (GAD-7).

Overall, these results indicate that students with chronic somatic disorders experience higher levels of stress, depressive symptoms and anxiety, and they use both professional mental health services and university support services more frequently than students without chronic somatic disorders. Awareness of available services, however, did not differ between the groups.

#### Group 3: Students diagnosed with a mental health disorder.

**Sociodemographic Characteristics:** In total, *n* = 1,239 (22.6%) students reported having been diagnosed with a mental disorder. The three most common mental disorders reported were depression (49.2%), anxiety (34.9%), and eating disorder (17.7%). Other diagnoses included ADHD (Attention deficit hyperactivity disorder) (7.7%), personality disorder (7.5%), OCD (obsessive-compulsive disorder; 6.7%), and bipolar disorder (2.4%). Three-quarters of these students identifies female (75.1%, *n* = 931), 20% (*n* = 248) identified as male, and 4.8% (*n* = 60) identified as gender diverse. The average age of students diagnosed with a mental disorder were *M* = 24.61 (*SD* = 4.9) years old, while students without a prior diagnosis had an average age of *M* = 23.45 (*SD* = 4.74) years old. Nearly one quarter (*n* = 305, 24.6%) of diagnosed students also reported having a chronic somatic disorder, compared to *n* = 628 (14.8%) the students without a mental disorder (*p* < .001).

Students that had previously been diagnosed with a mental disorder reported higher depressive symptoms, higher symptoms of stress, higher anxiety symptoms and less perceived social support than their fellow students not reporting a mental disorder diagnosis did (all *p* < .001). For details, see Table 3. The majority of students diagnosed with a mental disorder (98.0%) indicated that they would seek professional help if experiencing mental health problems, while 93.7% of their counterparts did (*p* < .001). Regarding past professional help-seeking behavior both group differed significant: while 92.4% of the students with a mental disorder already sought professional help in the past, only 27.6% of the other students did (p < .001). In relation to the students’ awareness of their universities help- and support services, both groups only differed in regards of their awareness of the psychosocial counseling (p < .001). Students diagnosed with a mental disorder took significant greater advantage of the following services in the past: student advisory service, psychosocial student counseling and workshops/seminars for university related stress and anxiety (all *p* < .001). Additionally, students diagnosed with a mental disorder showed a significant higher willingness to use individual counseling, group counseling and phone counseling and fewer willingness to use forums or blogs (all *p* < .001) compared to the students without a mental disorder. Students diagnosed with a mental disorder more frequently emphasized professional expertise, anonymity, short notice contact options, and free access as important features of an online mental health support program than their counterparts without a mental disorder (all *p* < .001) These findings had relatively small effect sizes. These findings indicate that students with a metal health disorder experience substantially higher levels of depression, anxiety and stress, and they report significant higher intention to seek help as well as higher past use of professional mental health services and university support services. Differences in service awareness between these groups are slight.

**Table 3:** Group comparison between students with a previous mental health disorder and students without a diagnosis in regards of user intention and actual usage of (mental health) support services.

Variable, *M (SD)*	Previously diagnosed students (*n* = 1,239)	Not previously diagnosed students (*n* = 4,235)	Test	*p*	Effect size
Psychopathology					
Stress (PSS4)	8.42 (3.28)	6.88 (3.08)	*F*(1, 5474)= 230.09	**<.001**	η² = .04
Social support (ESSI)	19.90 (4.33)	20.94 (3.99)	*F*(1, 5474)= 62.38	**<.001**	η² = .01
Depressive Symptoms (PHQ-9)	11.18 (5.99)	7.51 (5.07)	*F*(1, 5474)= 459.90	**<.001**	η² = .08
Anxiety (GAD7)	10.21 (4.96)	7.03 (4.49)	*F*(1, 5474)= 455.355	**<.001**	η² = .08
Variable, *n (%)*					
Gender, *n* (%)			χ^2^(2) = 134.9	**<.001**	ϕ = .16
Female	931 (75.1)	2,844 (67.2)			
Male	248 (20.0)	1,351 (31.9)			
Diverse	60 (4.8)	40 (0.9)			
Age, *M* (*SD*)	24.61 (4.9)	23.45 (4.74)	F(1, 5474)= 40.043	**<.001**	ϕ = .007
Intentions of use professional health care, *n* (%)	1,214 (98.0)	3,969 (93.7)	χ^2^(1) = 34.61	**<.001**	ϕ = -.08
Past use professional health care, *n* (%)	1,145 (92.4)	1,170 (27.6)	χ^2^(1) = 1648.5	**<.001**	ϕ = -.55
Past usage, student advisory service, *n* (%)	321 (25.9)	674 (15.9)	χ^2^(1) = 64.360	**<.001**	ϕ = **-**.12
Awareness, psychosocial student counseling, *n (%)*	929 (75.0)	2,522 (59.6)	χ2(1) = 97.93	**<.001**	ϕ = -.13
Past usage, psychosocial student counseling, *n (%)*	340 (27.4)	282 (6.7)	χ2(1) = 411.07	**<.001**	ϕ = -27
Past usage, seminars and workshops regarding stress, anxiety, *n (%)*	177 (14.3)	429 (10.1)	χ2(1) = 16.82	**<.001**	ϕ = -.06
Potential use of, individual counseling, *n (%)*	1,083 (87.4)	3,408 (80.5)	χ2(1) = 31.31	**<.001**	ϕ = -.076
Potential use of, group counseling, *n (%)*	350 (28.2)	939 (22.2)	χ2(1) = 19.66	**<.001**	ϕ = -.06
Potential use of, phone counseling, *n (%)*	345 (27.8)	965 (22.8)	χ2(1) = 13.48	**<.001**	ϕ = -.05
Potential use of, forums/ blogs, *n (%)*	284 (22.9)	1,171 (27.7)	χ2(1) = 10.98	**<.001**	ϕ = .05
Importance of expertise in an online-support offer, *n (%)*	1,109 (89.5)	3,636 (85.9)	χ2(1) = 11.07	**<.001**	ϕ = -.05
Importance of anonymity in an online-support offer, *n (%)*	857 (69.2)	3,194 (75.4)	χ2(1) = 19.47	**<.001**	ϕ = .06
Importance of short-notice contact option in an online-support offer, *n (%)*	1,079 (87.1)	3,485 (82.3)	χ2(1) = 15.91	**<.001**	ϕ = -.05
Importance of being for free*, n (%)*	1,094 (88.3)	3,482 (82.2)	χ2(1) = 25.82	**<.001**	ϕ = -.07

*Notes.* PSS-4: Perceived Stress Scale; ENRICHD Social Support Inventory (ESSI); Depressive symptoms were measured with Patient-Health-Questionnaire-9 (PHQ-9) and Anxiety was measured with Generalized Anxiety Disorder scale (GAD-7).

#### Group 4: Group comparison between students without a migration history, with a migration history, and international students.

In this group students without a migration history (*n* = 4,774, 87.2%), with a migration history (*n* = 393, 7.2%), and international students (*n* = 307, 5.6%) were compared in terms of their psychopathology, intention to seek help in a professional and university context and their actual past use of these services.

The students without a migration history had a mean age of *M* = 23.64 (*SD* = 4.85) years. Students with a migration history were *M* = 22.85 (*SD* = 3.79) years old, while the international students had a mean age of 25.8 (*SD* = 4.76) old. Across all three groups, gender was distributed similarly: the majority identified as female, about one third as male, and a small percentage as diverse (see Table 4 for details). The proportion of parental students was smallest among students with a migration history (8%), while 6% of students without a migration history and international students indicated that they had at least one child. Across all groups, over 20% of students received a psychological diagnosis, with students with a migration history reporting the highest percentage (26.7%), followed by international students (23.8%) and students without a migration history (22.2%). Regarding chronic somatic disorders, students with a migration history most frequently reported having such a condition (19.6%), compared to 17.2% of students without a migration history and 12.1% of international students. Concerning their psychopathological symptoms, the three groups differentiate as follows: the students without a migration history reported the lowest scores regarding stress, anxiety, and depression and the highest scores in their perceived social support. Students with a migration history reported similar scores in their perceived social support but higher stress and anxiety then the students without a migration history. The students with a migration history reported the highest PHQ-9 scores on the questionnaire for depression in the group. International students reported the lowest perceived social support and the highest levels of anxiety and stress symptoms. These findings were significant with small effect sizes (all *p* < .001).

**Table 4:** Group comparison between students with and without a migration history and international students.

Variable, *M (SD)*	Students without a migration history (*n* = 4,774, 87.2%)	Students with a migration history (*n* = 393, 7.2%)	International students (*n =* 307, 5.6%)	Test	*p*	Effect size
Psychopathology						
Stress (PSS-4)	7.12 (3.17)^a^	7.65 (3.28)^b^	8.33 (3.26)^b^	*F*(2, 5474) = 24.64	**<.001**	η² = .01
Social Support (ESSI)	20.89 (3.96)^a^	20.09 (4.29) ^b^	18.57 (5.11)^c^	*F*(2, 5474) = 51.65	**<.001**	η² = .02
Depressive Symptoms (PHQ-9)	8.14 (5.40)^a^	9.46 (5.65)^b^	8.34 (5.51)^b^	F(2, 5474)= 26.77	**<.001**	η² = .01
Anxiety (GAD-7)	7.62 (4.71)^a^	8.24 (5.05)^a,b^	9.16 (5.34)^b^	F(2, 5474)= 17.31	**<.001**	η² = .01
Variables, *n (%)*						
Gender, *n* (%)				χ^2^(4) = 66	.956	ϕ = .01
Female	3286 (68.8)	278 (70.2)	211 (68.7)			
Male	1401 (29.2)	108 (27.5)	90 (29.3)			
Diverse	87 (1.8)	7 (1.8)	6 (2.0)			
Age, *M* (*SD*)	23.64 (4.85)	22.85 (3.79)	25.83 (4.76)	F(1, 5474)= 56.384	**<.001**	ϕ = .010
Past use professional health care, *n* (%)	1,961 (41.1)^a^	187 (47.6)^b^	167 (54.4)^b^	χ^2^(2) =25.83	**<.001**	ϕ = .07
Awareness, student advisory service, *n* (%)	3,923 (82.2)^a^	315 (80.2)^a^	175 (57.0)^b^	χ^2^(2) = 117.01	**<.001**	ϕ = .15
Awareness, psychosocial student counseling, *n (%)*	3,045 (63.8)^a^	247 (62.8)^a^	159 (51.8)^b^	χ2(2) = 17.81	**<.001**	ϕ = .06
Awareness, counseling service for parental students, n (%)	3,351 (70.2)^a^	253 (64.4)^b^	138 (45.0)^c^	χ2(2) = 88.08	**<.001**	ϕ = .13
Awareness, seminars and workshops regarding stress, anxiety, *n (%)*	2,742 (57.4%)^a^	225 (57.3)^a^	131 (42.7)^b^	χ2(2) = 25.67	**<.001**	ϕ = .07
Awareness, orientation events, *n (%)*	3,205 (67.1)^a^	265 (67.4)^a^	174 (56.7)^b^	χ2(2) = 14.31	**<.001**	ϕ = .05
Awareness, digital offers, *n (%)*	2,207 (46.2)^a^	199 (50.6)^a^	115 (37.5)^b^	χ2(2) = 12.51	**.002**	ϕ = .05
Potential use of, literature, *n (%)*	1,781 (37.3)^a^	136 (34.6)^a^	70 (22.8)^b^	χ2(2) = 26.771	**<.001**	ϕ = .07
Potential use of, homepages, *n (%)*	2,190 (45.9)^a^	185 (47.1)^a^	84 (27.4)^b^	χ2(2) = 40.75	**<.001**	ϕ = .09
Potential use of, forums/ blogs, *n (%)*	1,303 (27.3)^a^	100 (25.4)^a^	52 (16.9)^b^	χ2(2) = 16.13	**<.001**	ϕ = .05
Importance of anonymity in an online-support offer, *n (%)*	3,581 (75.0)^a^	292 (74.3)^a^	178 (58.0)^b^	χ2(2) = 43.51	**<.001**	ϕ = .09
Importance of short-notice contact option in an online-support offer, *n (%)*	4,041 (84.6)^a^	314 (79.9)^b^	209 (68.1)^c^	χ2(2) = 60.82	**<.001**	ϕ = .12
Importance of reference person in an online-support offer, *n (%)*	3187 (66.8)^a^	261 (66.4)^a^	165 (53.7)^b^	χ2(2) = 21.80	**<.001**	ϕ = .06
Importance of being for free*, n (%)*	4,004 (83.9)^a^	337 (85.8)^a^	235 (76.5)^b^	χ2(2) = 12.716	**.002**	ϕ = .05

Notes. PSS-4: Perceived Stress Scale; ENRICHD Social Support Inventory (ESSI); Depressive symptoms were measured with Patient-Health-Questionnaire-9 (PHQ-9) and Anxiety was measured with Generalized Anxiety Disorder scale (GAD-7).

There were no significant group differences in willingness to seek professional help for mental health problems. However, actual past help-seeking behavior differed: only 41.4% of students without a migration history reported seeking professional help (e.g., from psychologists, psychiatrists, clinics, psychosocial counseling services, or online programs), compared to 47.6% of students with a migration history and 54.4% of international students (p < .001).

Regarding awareness of university help and support services, international students showed the least awareness across all service types. Students with and without a migration history only showed marginal differences in awareness (all p < .001). However, actual use of university support services did not differ significantly across the three groups.

In terms of user intention toward future help-seeking offers, there were no significant differences between groups for individual counseling, group counseling, or chat counseling. However, international students showed less interest in phone counseling, mail counseling, literature, forums, and websites compared to the other groups. Overall, students without a migration history reported the highest intention to use most of these services—except for websites, where students with a migration history reported the greatest intention to use them.

Across all groups, the most valued features of a potential online support program were expertise, the option for short-notice contact, and being free of charge.

Taken together, these results show that students without a migration history reported the lowest symptom levels, while international students report the highest levels of anxiety and stress and students with a migration history show the highest levels of depressive symptoms. Awareness of university support services was lowest among international students, and past use of professional mental health services was most common among these students.

#### Group 5: Group comparison between female, male and gender diverse students.

This group analysis compares female, male and gender diverse students regarding their awareness and help-seeking intentions towards professional and university support offers.. Two quarters of the students were female, and 23.5 (*SD* = 4.83) years old. Approximately 30% of the participants were male and just a small percentage of the participants identified as “gender divers” (1.8%). Male and gender diverse students did not differ regarding their age. Around 6% of female and gender diverse students reported being parents, compared to 4.8% of male students. Whereas 60% of the gender diverse students reported they received a mental health diagnosis prior to their participation, only 15.5% of the male students and 24.7% of the female students indicated the same. Regarding chronical, physical conditions the same muster prevails: 30% of gender diverse students reported that they are affected by a chronical physical condition, while 18.0% of the female and 13.9% of male participants reported the same.

In relation to their psychopathological symptoms the significantly highest level of anxiety (GAD-7), stress (PSS-4) and depression (PHQ-9) was reported by the gender diverse students, whereas their level of perceived social support was the lowest in the group comparison. The female university student’s results were in the middle between diverse and male students, while their perceived social support reached the highest scores. Male students reported the lowest symptoms of anxiety and depression and middle perceived social support. These findings were significant (all *p* < .001), for details, see Table 5 below. Asked for their potential and past professional help-seeking behavior (psychiatrist, psychotherapy, psychological/psycho-social counseling, inpatient treatment in a psychiatric/psycho-somatic clinic or online help services for mental health problems), the three groups differed significantly (both *p* < .001): the gender diverse students not only showed the significantly highest potential help-seeking intention (97.0%), but also reported the significantly highest actual use of professional help seeking services in the past (79.0%). The female students reported a significantly? higher intention of professional help seeking (95.9%) than their male fellow students (91.6%), which also reflects on their respective past use of professional help seeking behavior (female 45.8% versus 37.7% male). Regarding their awareness and user intention of university support offers, this trend continues: female and gender diverse students did not significantly differ in their awareness of university support offers, but gender diverse students used the student advisory service, the psychosocial counseling and workshops significantly more often than their female and male fellow students did (all *p* < .001). It is noticeable that the male students reported the lowest awareness regarding most university support offers (student advisory service, psychosocial counseling, counseling service for parental students and workshops) and used these services the least in comparison to the female and gender diverse students. Regarding the awareness and use of digital offers and the awareness of orientation days there was no difference between the three groups. The gender diverse and male students visited the orientation days less in comparison with the female students. Asked for their user-intention of university help-seeking offers the male students reported the least user-intention over all offers while the gender diverse students showed the greatest willingness to use the individual counseling, group counseling, phone-counseling and homepages, while the female students reported the highest motivation to use Chat-counseling, literature and forums. Female and gender diverse students showed equal user intention towards Mail-counseling. On average, the three groups put the highest value in a potential online program on expertise, the program being free, anonymity and the short notice option. In addition, the gender diverse students put the highest value towards a good user experience and no data-exchange between the program and the user’s health insurance.

**Table 5:** Group comparison of female, male and gender diverse students (*N* = 5,474).

Variable, *M (SD)*	Female students (*n* = 3,375, 69.0%)	Male students (*n* = 1,599, 29.2%)	Gender diverse students (*n =* 100, 1.8%)	Test	*p*	Effect size
**Psychopathology**						
Stress (PSS-4), *M* (*SD*)	7.43 (3,13)^a^	6.64 (3,25)^b^	9.19 (2.98)^c^	*F*(2, 5474) = 54.17	**<.001**	η² = .02
Social Support (ESSI), *M* (*SD*)	21.18 (3.78)^a^	19.70 (4,54)^b^	18.68 (4,4)^c^	*F*(2, 5474) = 88.79	**<.001**	η² = .03
Depressive Symptoms (PHQ-9), *M* (*SD*)	8.63 (5.56)^a^	7.34 (5.38)^b^	13.36 (5.61)^c^	*F*(2, 5474) = 74.73	**<.001**	η² = .03
Anxiety (GAD7), *M* (*SD*)	8.15 (4.74)^a^	6.60 (4.64)^b^	10.91 (5.10)^c^	*F*(2, 5474) = 83.16	**<.001**	η² = .03
Age, *M* (*SD*)	23.53 (4.83)	24.10 (4.72)	24.11 (25.01)	*F*(1, 5474) = 54.92	**<.001**	ϕ = .010
Variables, *N* (%)						
Intentions of use professional health care, *n* (%)	3.621 (95.9)^a^	1.465 (91.6)^b^	97 (97.0)^a,b^	χ^2^(2) = 42.36	**<.001**	ϕ = .09
Past use professional health care, *n* (%)	1.729 (45.8)^a^	507 (37.7)^b^	79 (79.0)^c^	χ^2^(2) =147.66	**<.001**	ϕ = .16
Awareness, student advisory service, *n* (%)	3.069 (81.3)	1.264 (79.0)	80 (80.0)	χ^2^(2) = 3.66	.160	ϕ = .03
Awareness, psychosocial student counseling, *n (%)*	2.489 (65.9)^a^	896 (56.0)^b^	66 (66.0)^a,b^	χ^2^ (2) = 47.62	**<.001**	ϕ = .09
Past usage, psychosocial student counseling, *n (%)*	453 (12.0)^a^	141 (8.8)^b^	28 (28.0)^c^	χ2(2) = 39.29	**<.001**	ϕ = .09
Awareness, counseling service for parental students, n (%)	2.670 (70.7)^a^	1.007 (63.0)^b^	65 (65.0)^a,b^	χ2(2) = 31.74	**<.001**	ϕ = .08
Awareness, seminars and workshops regarding stress, anxiety, *n (%)*	2.237 (59.3%)^a^	808 (50.5)^b^	53 (53.0)^a,b^	χ2(2) = 35.36	**<.001**	ϕ = .08
Past usage, seminars and workshops regarding stress, anxiety, *n (%)*	472 (12.5)^a^	120 (7.5)^b^	14 (14.0)^a,b^	χ2(2) = 29.40	**<.001**	ϕ = .07
Past usage, orientation events, n (%)	979 (25.9)^a^	321 (20.1)^b^	19 (19.0)^a,b^	χ2(2) = 22.53	**<.001**	ϕ = .06
Would potentially use, chat counseling, *n (%)*	1.525 (40.4)^a^	501 (31.3)^b^	35 (35.0)^a,b^	χ2(2) = 39.63	**<.001**	ϕ = .09
Would potentially use, literature, *n (%)*	1.477 (39.1)^a^	475 (29.7)^b^	35 (35.0)^a,b^	χ2(2) = 43.18	**<.001**	ϕ = .09
Would potentially use, homepages, *n (%)*	1.762 (46.7)^a^	647 (40.5)^b^	50 (50.0)^a,b^	χ2(2) = 18.58	**<.001**	ϕ = .06
Would potentially use, forums/ blogs, *n (%)*	1.084 (28.7)^a^	349 (21.8)^b^	22 (22.0)^a,b^	χ2(2) = 28.41	**<.001**	ϕ = .07
Importance of expertise in an online-support offer, *n (%)*	3.319 (87.9)^a^	1.339 (83.7)^b^	87 (87.0)^a,b^	χ2(2) = 17.02	**<.001**	ϕ = .06
Importance of anonymity in an online-support offer, *n (%)*	2.872 (76.1)^a^	1.108 (69.3)^b^	71 (71.0)^a,b^	χ2(2) = 27.36	**<.001**	ϕ = .07
Importance of short-notice contact option in an online-support offer, *n (%)*	3.251 (86.1)^a^	1.231 (77.0)^b^	82 (82.0)^a,b^	χ2(2) = 67.74	**<.001**	ϕ = .11
Importance of reference person in an online-support offer, *n (%)*	2.549 (67.5)^a^	992 (62.0)^b^	72 (72.0)^a,b^	χ2(2) = 16.69	**<.001**	ϕ = .06
Importance of being for free*, n (%)*	3.271 (86.6)^a^	1.217 (76.1)^b^	88 (88.0)^a^	χ2(2) = 92.41	**<.001**	ϕ = .13
Importance of no contact between insurance and an online-support offer, *n (%)*	2.233 (59.2)^a^	804 (50.3)^b^	67 (67.0)^b^	χ2(2) = 40.40	**<.001**	ϕ = .09

Notes. PSS-4: Perceived Stress Scale; ENRICHD Social Support Inventory (ESSI); Depressive symptoms were measured with Patient-Health-Questionnaire-9 (PHQ-9) and Anxiety was measured with Generalized Anxiety Disorder scale (GAD-7).

Combined, these results show that female students reported higher levels of stress, depressive symptoms and anxiety than male students, as well as higher perceived social support, greater awareness of university support services and more past use of mental health services and university support services. Gender-diverse showed elevated symptom levels and the highest past mental health service use.

The qualitative data analyses of this free text field explored the answers of *n =* 334 (6.1%) students, to grasp a better understanding of the students’ attitude towards help-seeking. Through the qualitative analysis process, eight main categories where formed: six main categories focusing on different approaches towards help-seeking (Social environment, professional care, spirituality, media, personal strategies and university help offers, one category for general comments (“The mental health supply situation is very bad”; “More time to study”), and one category capturing all answers that indicated that the students had no need for mental health support (“I don’t have problems”).

In total, 23 sub-categories were formed. The six main categories with specific answers on potential help-seeking (i.e., social environment, professional care, spiritual, personal strategies, media, and university help offers) had each between two and six subcategories, on average each main category had 3.8 subgroups, as presented in Table 6. The three most answered categories were 1. social environment (39.5%), 2. professional care (21.0%) and 3. spirituality (20.1%).

**Table 6:** Results of the qualitative data analyses of the free text field: “What kind of service would you use, in case of mental health problems.

Categories and sub-categories, n (%)	Students (*N* = 334)	Examples of free text field answers
**1. Social environment**	132 (39.5%)	
**Social environment (general)**	75 (22.5%)	“Support through social environment”
**Friends**	35 (10.5%)	“Talking to my friends”
**Family**	21 (6.3%)	“Parents”
**Pets/ Animals**	1 (0.3%)	“Cats/animals”
**2. Professional care**	70 (21.0%)	
**Self-help-group/ contact with others concerned**	33 (9.9%)	“Self-help group”
**Professional care (General answers)**	25 (7.5%)	“Group therapy”
**GP (general practitioner)**	5 (1.5%)	“My first contact would be my general doctor, who I trust very much”
**Psychotherapy/ trauma therapy**	4 (1.2%)	“Trauma specialist, EMDR therapy”
**Coaching**	3 (0.9%)	“Privately paid coaching”
**3. Spirituality**	67 (20.1%)	
**Christian counseling**	37 (11.1%)	“Counseling through my pastor”
**Prayer/ Faith/ God**	10 (2.9%)	“Praying to god”
**Church/ Pastor**	8 (2.4%)	“Pastor”
**Meditation**	7 (2.10)	“Meditation, positive affirmations, yoga”
**Shaman**	2 (0.6%)	“Shaman”
**Religious literature**	1 (0.3%)	“Religious materials/ motivational books”
**4. Media**	18 (5.4%)	
**Podcast**	10 (3%)	“Podcast on psycho-education, meditation”
**Web resources, YouTube**	8 (2.4%)	Healthy GamerGG”
**5. Personal strategies**	17 (5.1%)	
**Personal strategies (general)**	8 (2.4%)	“Hard work alone”
**Sport/ Yoga**	4 (1.2%)	“Sport as resilience pusher”
**Hobbies**	3 (0.9)	“Travel to the peaceful countryside”
**Drug consumption**	2 (0.6%)	“Heroin”
**6. University help offers**	11 (3.3%)	
**Psychosocial Counseling**	6 (1.8%)	“University offers for counseling”
**Workshops**	5 (1.5)	“Dresden frühdran” (specific name of a workshop offered by TU Dresden)
**7. General comments**	11 (3.3%)	“Mental health supply situation is very bad”
**8. No mental Health problems/ no help required**	8 (2.4%)	I don´t need offers, as I don´t have mental health problems”

## Discussion

The aim of this paper was to gain a deeper understanding of the mental health status, help-seeking intentions, and help-seeking behavior of particular at-risk groups among university students. The results of this study highlight the current socio-demographic diversity, with some student subgroups being more at risk for mental health problems than others. Historically, a hundred years ago, the typical student was male, young, and from a prosperous family. Times have changed, and demographic shifts have opened access to universities, but individual challenges remain: high financial burden, stress, childcare, academic struggles, to name a few [19]. As diverse as today’s student body is, so are their individual life challenges. Interpreting these findings within Andersen`s Model of Health Service Use allows for a differentiated understanding of how predisposing, enabling and need-related factors interact in relation to mental health outcomes and help-seeking behavior among at-risk university student groups.

The quantitative analyses indicated that across all five group comparisons, students belonging in most predefined at-risk groups showed worse mental health outcomes (depressive symptoms, stress, anxiety, and social support) than their respective comparison groups. Only parental students showed a different pattern, with lower psychopathology and higher perceived social support than non-parents.

Across all five at-risk subgroups, participants showed a strong willingness to seek professional help in case of mental health problems. Some students showed high symptoms of mental health struggles (depression, anxiety, and stress), but a treatment gap exists between their awareness of university and professional mental health support offers and their actual use of these services. As stated above, surprisingly, parental students reported better mental health scores (significantly lower stress, anxiety, and depression) than non-parental students. There was no difference in perceived social support between groups. Given that parental students carry the responsibility of raising children alongside university commitments, these findings are rather unexpected. Other studies have shown contradictory trends [20,41], raising the question of why parenthood could be viewed as a protective factor for good mental health among university students in this study. As literature shows both risk and protective roles for parenthood regarding mental health, it could be interpreted that parenthood is a determinant that affects mental health depending on individual life circumstances. Within Andersen`s Model of Health Service Use, the lower symptom burden among parental students indicates reduced need factors, while parenthood may be understood as a context-dependent predisposing factor. The largely comparable awareness and use of support services suggest no substantial differences in enabling factors compared to non-parental students.

Parental students had used the student advisory service and, as expected, the parental advisory service significantly more often than non-parents.

Regarding students with a chronic somatic disorder, results showed that while they reported high levels of stress and psychopathology and thus had a higher need for support, they also used the university’s student advisory service and psychosocial counselling offers more often than students without a chronic somatic disorder.

Interpreted within Andersen`s Model of Health Service Use, this pattern indicates elevated need factors alongside accessible support services, highlighting students with chronic somatic disorders as an important target group for mental health services at universities.

Over 20% of the participants reported a prior mental health diagnosis, reflecting the need for university and professional mental health support services. In line with this finding, three consecutive student surveys showed a worrying trend [5]: in 2020, 2021, and 2022, levels of depression and suicidality continually rose, even though the pandemic was over and could no longer be held accountable as the main factor for students’ mental health struggles. In the present study, students with a previous mental health diagnosis differed strongly from those without: not only did they report significantly lower social support and higher anxiety, stress, and depression symptoms, but they also showed significantly higher intention to seek help and past use of support services. These students used the student advisory service, psychosocial counseling, and workshops significantly more often than those without a prior diagnosis. Viewed through Andersen`s Model of Health Service Use, this pattern reflects elevated need factors, accompanied by access to and use of available support services, resulting in increased help-seeking behavior.

Male mental health is a topic on its own. Male students showed the lowest intention to seek help and the lowest actual use of help-seeking services, while at the same time reporting the lowest scores on measures of depression (PHQ-9), anxiety (GAD-7), and stress (PSS-4).

In contrast, students who selected “diverse” as their gender option reported the highest levels of stress, anxiety and depression. Notably, 60% of these students had already received a mental health diagnosis. In addition, gender-diverse students reported lower perceived social support than other gender groups.

Interpreted within Andersen`s Model of Health Service Use, these findings suggest markedly elevated need factors among gender-diverse students, which are accompanied by comparatively high use of professional and university-based support services. This pattern indicates that, despite available services, existing support structures may be insufficient to adequately address the needs of this group. In contrast, lower help-seeking intentions of the male students may be related to predisposing factors, such as gender-specific norms and attitudes toward mental health and help-seeking [42,43].

Taken together, these findings indicate that the mental health of university students belonging to gender minorities in our sample is alarmingly poor, underscoring the urgent need to create targeted, gender-sensitive support services. Our findings highlight the necessity for further research to better understand the specific challenges faced by these students and to address broader societal issues such as discrimination and stigmatization within higher education settings. The need for counseling and support can vary significantly between different genders [44].

Compared to their peers without a migration history, international students and those with a migration history experienced greater stress (PSS-4), reported less social support (ESSI), and exhibited higher levels of depressive (PHQ-9) and anxiety symptoms (GAD-7). Interpreted in line with Andersen’s Model of Health Service Use, these findings indicate elevated need factors among these groups, while at the same time pointing to limitations in enabling factors, such as language barriers, differences in mental health literacy, stigma. However, further research is needed to better understand the mechanisms underlying this elevated psychological burden, including the potential detrimental impact of racism and xenophobia [45]. These findings highlight the importance of addressing the specific counseling and support needs of students with a migration history and international students through tailored and accessible support services.

The qualitative data analysis provided valuable insights and surprising results regarding potential help-seeking among university students. Despite the free text field being included in the professional help-seeking part of the questionnaire, almost 40% of the students indicated they would primarily seek help in their direct social environment rather than in a professional context. Some students specified family or friends, whereas others used the broader term “social environment.” “Social environment” was the category with the most responses, highlighting the importance of and strong reliance young people place on friends and family for support during mental health struggles. Relatedness is a basic human need according to Deci and Ryan’s Self-Determination Theory [46], and the results of the qualitative data analysis reflect that need.

The second main category was “professional care.” Answers in this category named specific points of contact, such as “trauma specialist,” “family doctor,” and “coaching.” Interestingly, the most frequent answers belonged to the subcategory “self-help group/contact with others concerned” (9.9%), reflecting findings on the relevance of peer-to-peer support in mental health crises [47]. Combining the insights from both qualitative categories reveals a coherent pattern: social connectedness emerges as a central need across students’ support preferences. In the first category, students most frequently expressed a desire for emotional and relational support, while in the second category self-help groups were among the most commonly mentioned preferred formats. This convergence suggests that need factors, as described in Andersen’s Behavioral Model, may manifest primarily through a desire for peer connection rather than through professional care. Universities may therefore benefit from expanding structured, socially oriented support opportunities that foster belonging, shared experience, and low-threshold peer interaction.

Another highly relevant and interesting finding of the qualitative data analysis was the strong religious orientation of the answers (20.1%), grouped under the main category “spiritual answers.” Especially notable was the frequency of the answer “Christian counselling” (11.1%). Other answers included the church in general, prayer, faith, and contact with a pastor. In times when polycrises [15] strain young people’s mental health, could a turn to religion serve as an anchor for students facing uncertainty and fear of the future? On the other hand, the connection between adolescence and religion has long been discussed in the literature (Josephson & Dell, 2004).

Main category four (“Media”) included answers regarding podcasts and information research through homepages, which was less than previously anticipated given that young people and adolescents are known for their high (social) media affinity [48]. In main category five (“Personal strategies”), all answers that showed students had a clear, individual strategy for dealing with mental health problems were included. These strategies involved actions the students could perform independently, without support from others (in contrast to main category one “Social environment”). Main category five showed a wide range of personal strategies, from “writing therapy” to “spending time in nature alone.” Category six included all answers naming university mental health initiatives, services, and workshops (specific or general).

Together, these categories reflect the broad spectrum of resources and strategies students draw upon in times of crisis, ranging from interpersonal support to individual coping strategies and institution-based offerings.

### Strengths and limitations

A major strength of this study lies in the in-depth exploration of students’ awareness, attitudes, and acceptance of existing professional and university support offers for mental health problems across different at-risk subgroups. Combining quantitative and qualitative data analyses provides valuable insight into students’ attitudes toward various potential offers, which can greatly assist in planning and implementing new mental health programs for students. Another strength is the large sample size and diversity of participants. Studies on students’ mental health often focus on students from specific fields (e.g., medical, nursing, or psychology students [15,49,50], whereas this study included students from six Saxonian universities, covering eight faculties overall [5]. For interpreting the results, it is important to note that group assignments were intersectional; participants could belong to more than one group, which presents a methodological limitation. A further limitation concerns the assessment of prior help-seeking. Although the questionnaire asked whether students had used professional mental health services either before or within the last year, it did not capture more detailed information about the timing, frequency, or context of previous service use. This restricts conclusions about how earlier help-seeking experiences may have shaped student’s current attitudes and preferences.

## Conclusion

In conclusion, the results of this study showed that students belonging to at-risk groups—specifically students with a chronic somatic disorder, those diagnosed with a mental health disorder, students with a migratory background/international students, and students with diverse gender identities—reported elevated levels of stress and mental health problems. Interpreting these findings through Andersen’s Behavioral Model suggest that heightened psychological distress reflects increased need factors, whereas differences in social support indicate variations in enabling factors that may shape help-seeking patterns.

In light of these findings, the potential of online programs to promote students’ mental health stands out as an excellent option to support students in their individual life circumstances. A major advantage of online programs and digital mental health interventions lies in their high flexibility: they can be accessed anytime and anywhere while incurring relatively low financial costs. University students are an ideal target audience for low-threshold online interventions, as they generally possess technical expertise, have limited free time, and often commute between their hometowns and university cities. Their vulnerability to developing mental health problems further supports their eligibility.

Given the results of this study and the sociodemographic diversity among students, it is clear that online interventions can be extremely helpful, as they can be tailored to various life circumstances and translated into different languages. International students can benefit from online interventions as much as students with physical or mental challenges, as well as parental students managing family responsibilities alongside their studies. A recent publication [51] showed that 45–55% of participating students expressed willingness to engage in e-mental health offers. Online interventions like the one in this study can fill the need for professional, personal, and educational mental health care and should be prioritized.

At the same time, the importance of traditional, face-to-face university help offers—such as the student advisory service and psychosocial counseling—is clear, as these services are widely known among participants and particularly used by at-risk groups like gender-diverse students, students with chronic somatic disorders, and students with mental health diagnoses. The study showed that across five subgroups, individual counseling was strongly favored over group counseling. This finding can provide useful guidance for designing future interventions.

Overall, the results provide insights for those working with student mental health services and those in stakeholder positions. The study offers deep insight into university students’ mental health help-seeking attitudes and behavior, as well as differences in mental health across at-risk subgroups. The findings underscore the urgent need to promote mental health among adolescents in universities and serve as important input for developing new guidelines and policymaking in higher education institutions. A recommended course of action for all involved is the regular assessment of the needs and mental health status of university students.
